# Comparison of Improvements in Quality of Life With Disease-Modifying Anti-rheumatic Drugs in Seropositive and Seronegative Rheumatoid Arthritis

**DOI:** 10.7759/cureus.100094

**Published:** 2025-12-25

**Authors:** Avinash Muraleedharan, Shweta Chaubey, Shalu A, Ashish Yadav

**Affiliations:** 1 Physical Medicine and Rehabilitation, All India Institute of Medical Sciences, Raipur, Raipur, IND; 2 Physical Medicine and Rehabilitation, National Institute for Locomotor Disabilities, Baranagar, IND; 3 Physical Medicine and Rehabilitation, Bangalore Baptist Hospital, Bengaluru, IND; 4 Physical Medicine and Rehabilitation, All India Institute of Medical Sciences, Nagpur, Nagpur, IND

**Keywords:** das28 esr, disease-modifying antirheumatic drugs (dmards), pain on vas, seronegative rheumatoid arthritis, sf-36: short form - 36

## Abstract

Background: Rheumatoid arthritis (RA) is a chronic, progressive autoimmune disorder characterized by joint pain, stiffness, and deformities, leading to functional impairment and reduced quality of life (QoL). Disease-modifying anti-rheumatic drugs (DMARDs) are the cornerstone of therapy, effectively reducing disease activity and improving health-related QoL (HRQoL).

Objective: To compare HRQoL outcomes between seropositive and seronegative RA patients and to assess the improvement in HRQoL following initiation of conventional synthetic DMARDs (csDMARDs) in both seropositive and seronegative RA patients.

Methodology: This prospective, comparative, non-randomized experimental study was conducted over 16 months and included 104 patients with RA who met the inclusion criteria - 87 seropositive and 17 seronegative. All participants received csDMARD therapy and were evaluated using the visual analogue scale (VAS) for pain, Disease Activity Score 28 (DAS28 ESR) for disease severity, and the SF-36 questionnaire for HRQoL at baseline, three months, and six months.

Results: Both seropositive and seronegative groups showed significant improvement in HRQoL after csDMARD initiation as measured by the SF-36 index. No significant intergroup differences were observed in HRQoL outcomes. However, the seronegative group demonstrated a more pronounced reduction in pain and disease severity, as indicated by VAS and DAS28-ESR scores, compared to the seropositive group.

Conclusion: Early initiation of csDMARDs in RA patients leads to significant improvement in QoL, regardless of serostatus. While both groups benefited similarly in HRQoL, seronegative patients experienced greater reductions in pain and disease activity, underscoring the efficacy of csDMARDs as an initial treatment strategy in RA management.

## Introduction

Rheumatoid arthritis (RA) is a chronic autoimmune disease characterized by joint pain, stiffness, and deformity in multiple regions, particularly the hands and feet [1]. Individuals with RA will have varying degrees of physical impairment, fatigue, fever, reactive depression, and weight loss [2]. The progression of RA becomes a burden to the patients, their families, and society because of the treatment costs, multiple hospital visits, and medical and surgical interventions. Work disability can occur due to increased functional impairment as the disease progresses.

The two serum biomarkers that are used widely in the classification and prognosis of RA are rheumatoid factor (RF) and anticitrullinated protein antibodies (ACPA). RF is an autoantibody that targets the fragment crystallizable portion of immunoglobulin G. ACPA are antibodies that target peptides and proteins containing citrulline. ACPA are as sensitive as RF, but more specific to RA [3], and they are detected earlier in RA [4]. The seropositive RA (SPRA) and seronegative RA (SNRA) subgroups were recognized after the identification of RF and ACPAs. The SPRA group was positive for one or both of ACPA and RF antibodies, while the SNRA group was negative for both.

The 2010 American College of Rheumatology (ACR)/European League Against Rheumatism (EULAR) classification criteria have been helpful in facilitating the earlier diagnosis of RA patients [5]. It redefined the population of RA and the classification of SNRA. A more severe disease course is seen in patients with SPRA who share certain environmental and genetic risk factors [6]. There is very little research about SNRA, but they also show some evidence of genetic associations [7]. There are conflicting studies about the clinical presentation and disease course of SNRA, which are reported as less severe than SPRA [8]. SNRA has been found to present with a less severe disease activity than SPRA, with radiographic damage being less [9]. Some studies report SPRA patients to have severe disease with functional impairment during the presentation and after treatment with disease-modifying anti-rheumatic drugs (DMARDs), while other studies report SNRA patients to have more inflammatory activity and poor radiological outcome [10-12].

The primary treatment objectives for RA are to reduce pain and to maintain physical and social function. The cornerstone of treatment for RA is DMARDs. They target pathways of inflammation responsible for joint swelling and damage. Conventional synthetic DMARDs (csDMARDs) are derived synthetically and do not have a specific molecular target, but are found to have activity in the treatment of RA. The preferred CsDMARD is methotrexate (MTX) because of its excellent benefit-to-toxicity profile [13]. The most commonly used CsDMARDs in combination with MTX are hydroxychloroquine, sulfasalazine, and leflunomide. Intra-muscular gold, cyclosporine, and azathioprine are less commonly used CsDMARDs. The 2023 EULAR recommendations suggest MTX to be part of the first-line treat-to-target strategy as the anchor drug unless contraindicated or not tolerated. A short course of glucocorticoids may be used as a bridging therapy when and where necessary. With this line of management, the aim of attainment of the target is six months, after which biologic DMARDs or targeted synthetic DMARDs are recommended [13].

DMARDs, which are derived through biologic processes and are designed to target specific cells or proteins involved in the inflammatory response, are called biologic DMARDs. The most recent class of medications approved for use in RA is the targeted synthetic DMARDs, which are developed through synthetic methods and designed to target specific cellular processes. Tofacitinib is the first targeted synthetic DMARD used for the treatment of RA.

RA has major, diverse effects on patients' health-related quality of life (HRQoL), spanning both physical and mental domains of well-being. Measurement of HRQoL is important for many reasons. Firstly, many patients value HRQoL more than disease-related variables, such as inflammatory biomarkers or joint counts. Secondly, reduced HRQoL in RA patients is associated with increased use of healthcare resources and with increased levels of depression. Therefore, the key therapeutic goal should be to limit the adverse effects of RA on HRQoL. DMARDs are found to be very effective in the remission of RA. Most of the studies done in the past focus on the radiological and treatment outcomes of SPRA or SNRA patients. Not many studies have been done comparing the improvement in HRQoL in patients with SPRA and SNRA. Thus, this study mainly focuses on the quality-of-life aspect of patients affected by RA and its improvement.

## Materials and methods

Aims and objectives

The primary aim of this study was to compare the outcomes of HRQoL between seropositive and seronegative patients diagnosed with RA. In addition, the study sought to assess the degree of improvement in HRQoL among these patients before and after the initiation of disease-modifying antirheumatic drugs (DMARDs). Through these objectives, the study intended to evaluate both intergroup differences and longitudinal changes in patient-reported outcomes associated with RA management.

Methods

This is a prospective comparative non-randomized observational study conducted in the outpatient department of Physical Medicine & Rehabilitation of an apex central institute. The study was approved by the ethics committee (ECR/1210/Inst/WB/2019) and performed as per the World Medical Association (WMA) Declaration of Helsinki. Cases were selected as per the inclusion and exclusion criteria.

A total of 104 patients with a diagnosis of RA, visiting the Physical Medicine & Rehabilitation OPD of the National Institute for Locomotor Disabilities, Kolkata, India, were included in the study. Out of these 104 patients, 87 patients were in the SPRA group, and 17 patients were in the SPRA group.

The inclusion criteria of the study were (1) a diagnosis of RA of one year or less according to the 2010 EULAR criteria and (2) no previous treatment with DMARDs.

Exclusion criteria were (1) patients younger than 18 years and older than 60 years; (2) patients already taking DMARDs at the time of first visit; (3) patients with undifferentiated arthritis not meeting 2010 EULAR criteria for RA; (4) non-inflammatory arthritis; (5) patients with any systemic infection; (6) pregnancy; (7) allergy to steroids, NSAIDs, and DMARDs; (8) immunocompromised patients; (9) patients with uncontrolled diabetes; (10) patients with severe cardiovascular, respiratory, renal, and hepatic diseases; and (11) bleeding diathesis and malignancy.

All the patients were advised to do the blood investigations and return with the reports. After obtaining satisfactory reports, csDMARDs were started along with a short course of glucocorticoids (when necessary), nonsteroidal anti-inflammatory drugs (NSAIDs) for pain relief, and exercise therapy, considering the first day as baseline. The same csDMARDs regimen consisting of methotrexate, sulphasalazine, and hydroxychloroquine, and a standardised exercise protocol was started for both groups. The DAS28 ESR score was calculated based on the number of tender joints, number of swollen joints, erythrocyte sedimentation rate (ESR), and the subjective assessment of disease activity by the patient during the preceding seven days on a scale between 0 and 100, with 0 being no activity and 100 being the highest possible activity [14]. A visual analogue scale (VAS) was used to measure the patient’s and physician’s assessment of disease activity [15]. The 36-item Short-Form Health Survey 1.0 (SF-36) developed by RAND was given to all the patients [16]. They were asked to fill out the questionnaire (tick boxes) by themselves and return it. The patients from both groups were then called for follow-up after three and six months, with the blood investigations done prior to the visit. The DAS28 ESR score, VAS score, and SF-36 questionnaire data were obtained during each visit. Data of each patient were entered on the study proforma.

Statistical analysis

All continuous variables were presented as mean±SD or median (IQR) as appropriate and were compared between the groups by independent t-test for normally distributed data and by the Mann-Whitney U test for non-normal data. All qualitative data were presented as numbers and percentages. Chi-square test or Fisher’s exact test was used to see the difference between the groups for qualitative variables. A p-value <0.05 was considered statistically significant. Statistical software Statistical Product and Service Solutions (SPSS, version 26; IBM SPSS Statistics for Windows, Armonk, NY) was used for data analysis.

## Results

The comparison of the outcomes of health-related quality of life between Seropositive and Seronegative patients and the assessment of improvement in HRQoL in RA patients before and after starting DMARDs were done under this study.

There were 87 patients in the SPRA group, and 17 patients in the SNRA group. The study duration was from January 2021 to May 2022. Demographic features of the patients are listed in Table 1.

**Table 1:** Demographic characteristics SPRA: Seropositive rheumatoid arthritis; SNRA: Seronegative rheumatoid arthritis

Parameter	SPRA (n=87)	SNRA (n=17)	Test statistic & P-value
Age	44.01±8.38	46.23±9.99	t = 0.99, p = 0.33
Sex Ratio (M:F)	27:60 (31%:69%)	5:12 (29.4%:70.6%)	χ² = 0.02, p = 0.89
Body Mass Index	26.2±2.55	25.91±3.03	t = 0.43, p = 0.67

**Figure 1:**
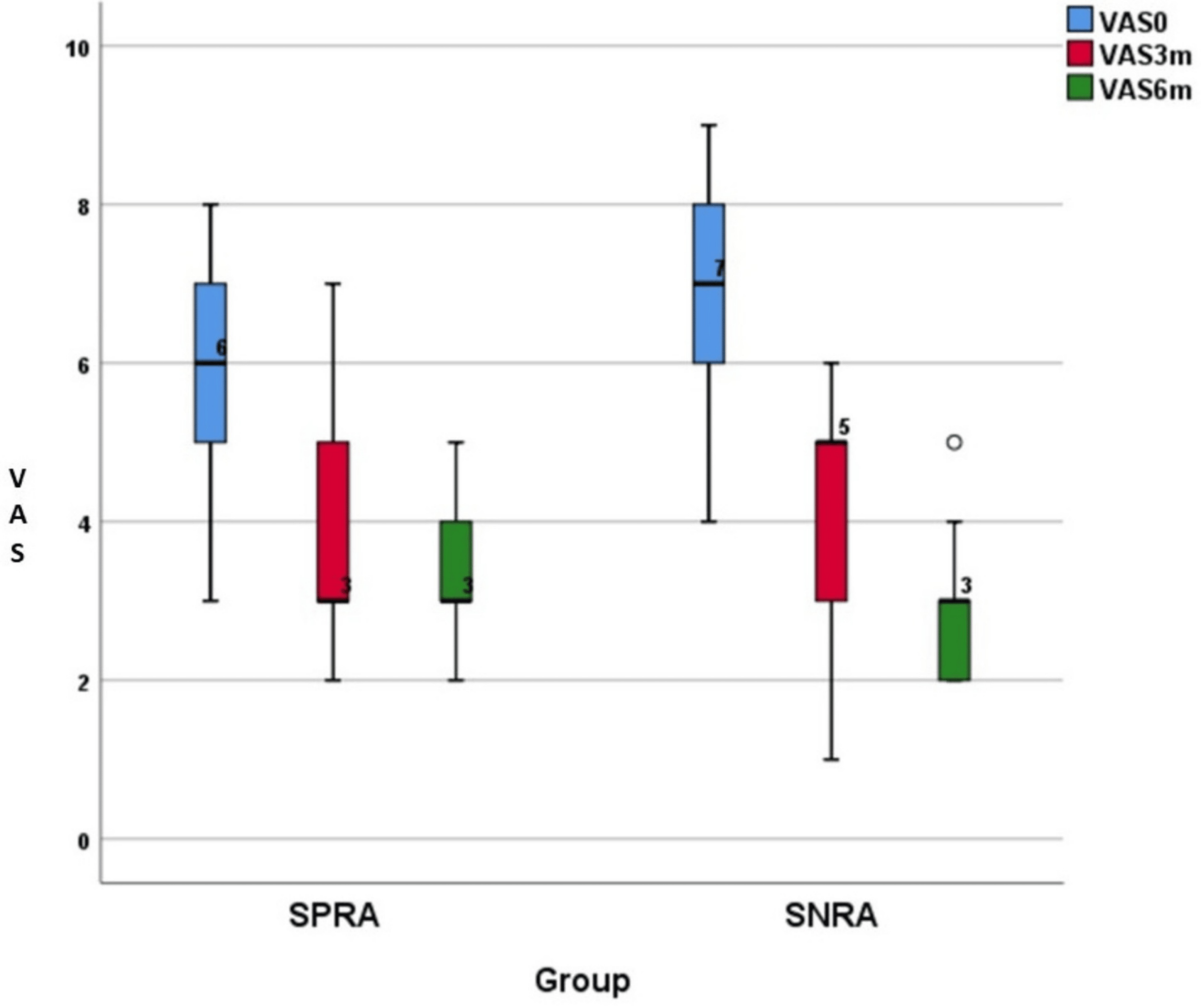
VAS at baseline, three months, and six months VAS: Visual analogue scale; SPRA: Seropositive rheumatoid arthritis; SNRA: Seronegative rheumatoid arthritis

**Figure 2:**
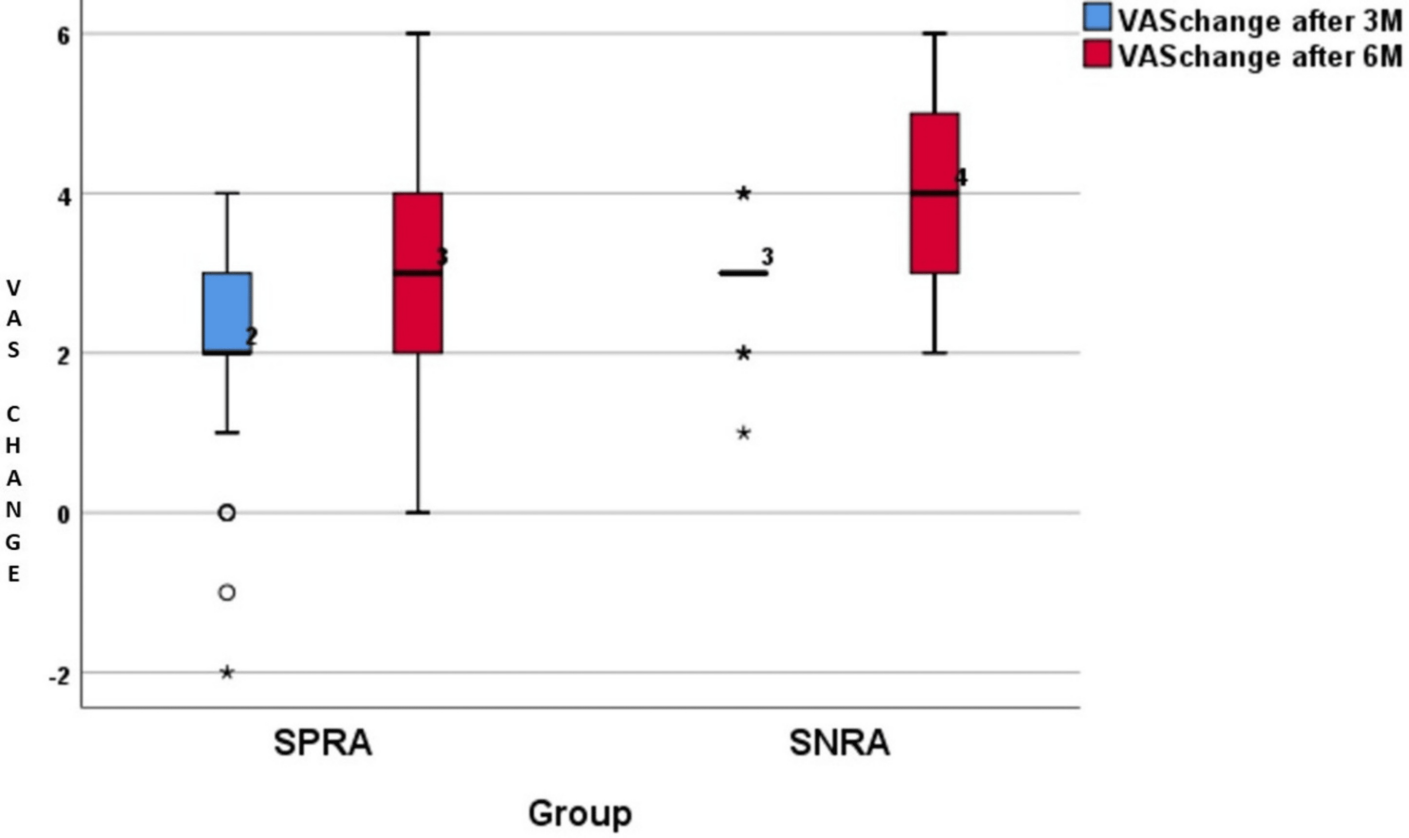
Difference in VAS scores after three months and six months DAS28 ESR: Disease activity score 28 erythrocyte sedimentation rate; SPRA: Seropositive rheumatoid arthritis; SNRA: Seronegative rheumatoid arthritis

The distribution of changes in the VAS is presented in Table 2 and illustrated in Figures 1-2. Pain scores were studied by using the VAS score. The baseline values of VAS were higher in the SNRA group compared to the SPRA group, which was statistically significant (p=0.003). This showed that pain was higher in the SNRA group compared to the SPRA group during the first visit. Table 2 shows that, at baseline, though pain was higher for SNRA than SPRA (p=0.003), at three and six months, changes in the score were statistically greater than those of the SPRA group (respective p-values were 0.01 and < 0.0001).

**Table 2:** Distribution of visual analogue scale changes SPRA: Seropositive rheumatoid arthritis; SNRA: Seronegative rheumatoid arthritis

Visual Analogue Scale	SPRA (n=87), median (IQR)	SNRA (n=17), median (IQR)	Test statistic & P-value
Baseline	6 (5,7)	7 (6,8)	U = 408.5, p = 0.003
3 months	3 (3,5)	5 (3,5)	U = 561.5, p = 0.11
6 months	3 (3,4)	3 (2,3.5)	U = 795, p = 0.6
Difference at 3 months	2 (2,3)	3 (2.5,3)	U = 470.5, p = 0.01
Difference at 6 months	3 (2,4)	4 (3,5)	U = 332.5, p = <0.0001

**Figure 3:**
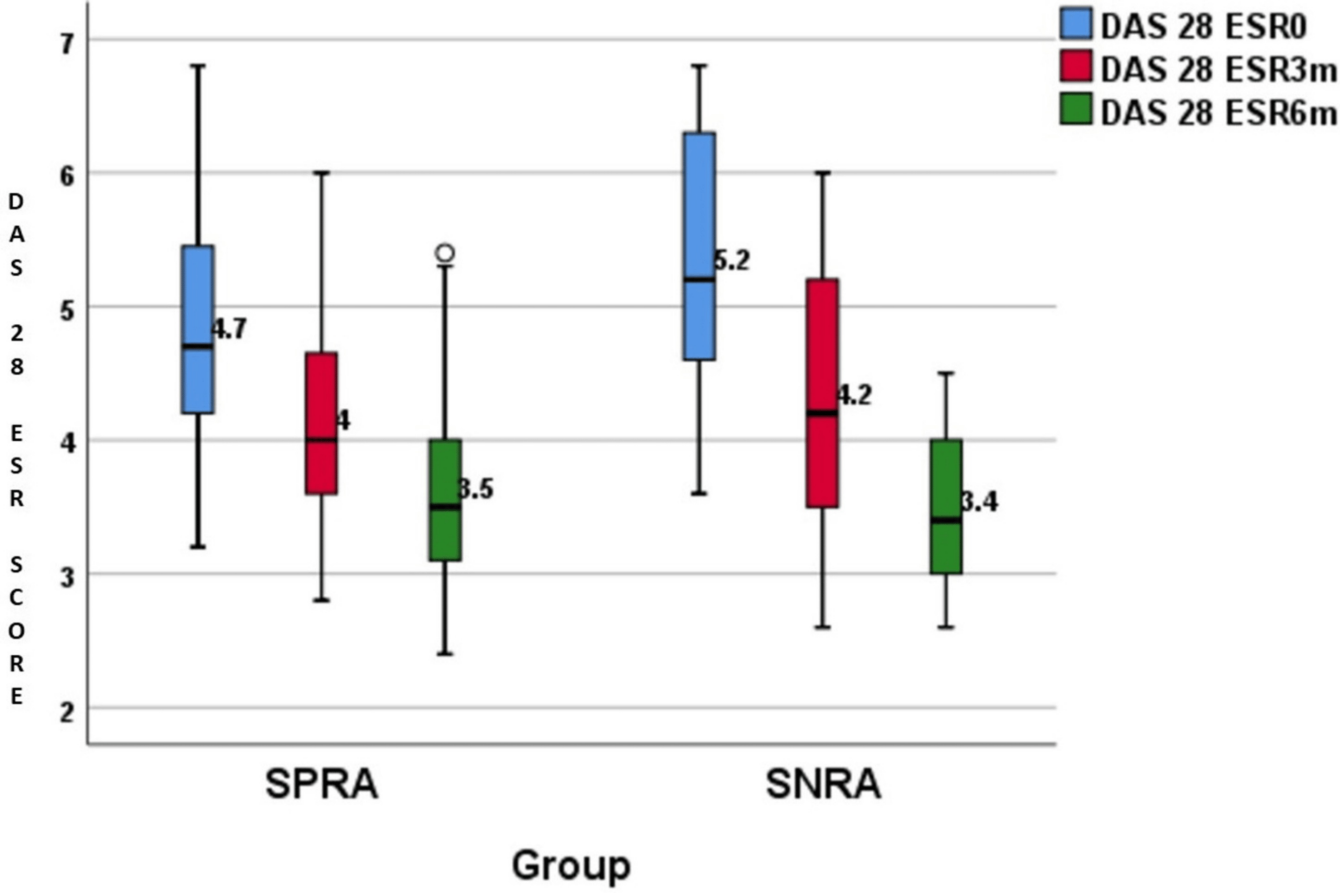
DAS28 ESR scores at baseline, three months, and six months DAS28 ESR: Disease activity score 28 erythrocyte sedimentation rate; SPRA: Seropositive rheumatoid arthritis; SNRA: Seronegative rheumatoid arthritis

**Figure 4:**
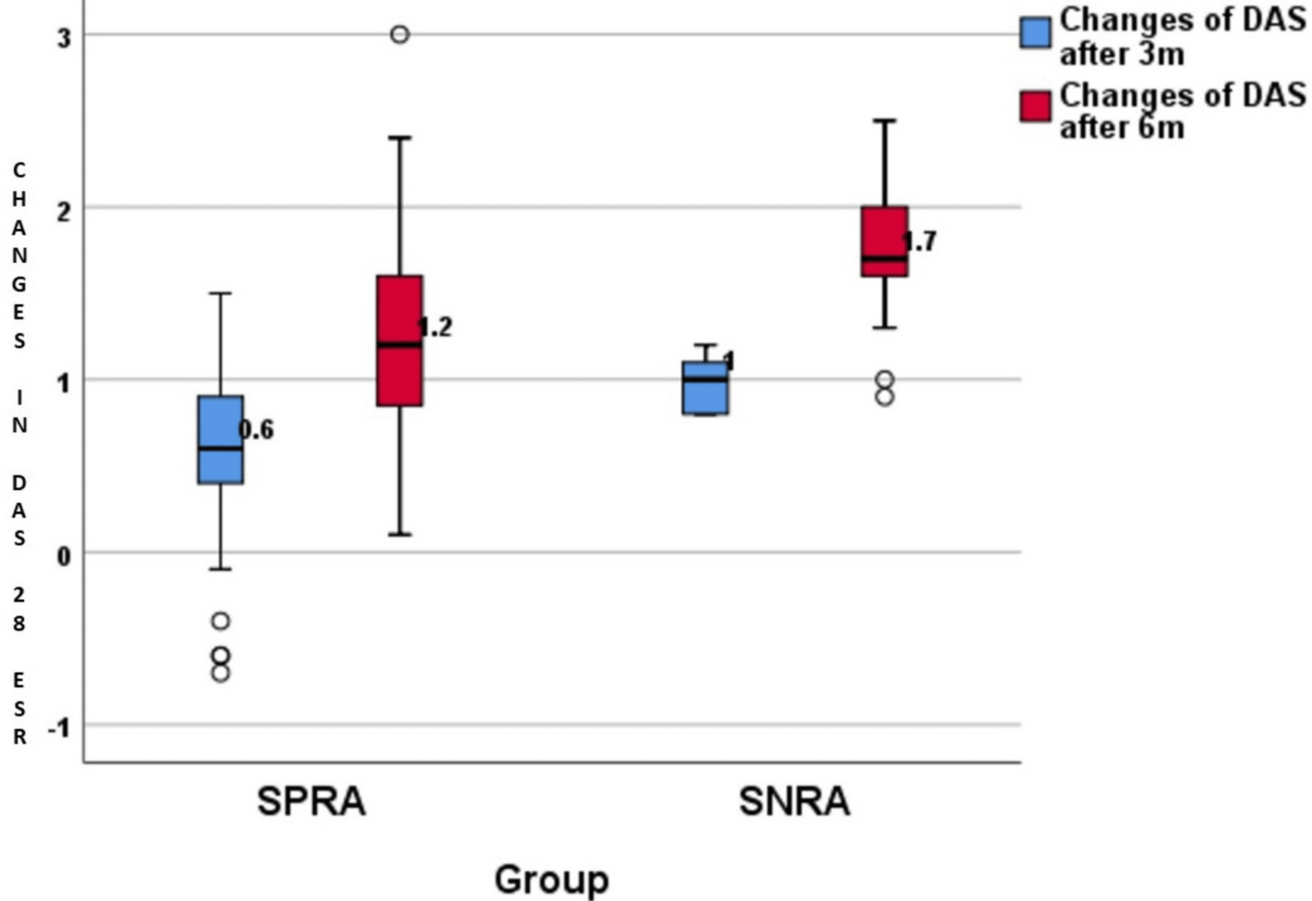
Changes in DAS28 ESR scores after three months and six months DAS28 ESR: Disease activity score 28 erythrocyte sedimentation rate; SPRA: Seropositive rheumatoid arthritis; SNRA: Seronegative rheumatoid arthritis

The distribution of DAS28 ESR score changes is presented in Table 3 and illustrated in Figures 3-4. The disease severity of RA was measured using DAS28 ESR scoring. Table 3 showed that the DAS28 ESR scores were almost similar between the SPRA and SNRA groups during the time period. However, it was interesting to note that the difference in DAS28 ESR scores at three months and six months was significantly bigger in SNRA (p-value<0.0001).

**Table 3:** Distribution of DAS28 ESR score changes DAS28 ESR: Disease activity score 28 erythrocyte sedimentation rate; SPRA: Seropositive rheumatoid arthritis; SNRA: Seronegative rheumatoid arthritis

DAS28 ESR	SPRA (n=87), median (IQR)	SNRA (n=17), median (IQR)	Test statistic & P-value
Baseline	4.7 (4.2,5.5)	5.2 (4.5,6.35)	U = 521.5, p = 0.055
3 months	4 (3.6,4.7)	4.2 (3.5,5.3)	U = 687 p = 0.64
6 months	3.5 (3.1,4)	3.4 (3,4.2)	U = 811, p = 0.53
Difference at 3 months	0.6 (0.4,0.9)	1 (0.8,1.15)	U = 334.5, p = <0.0001
Difference at 6 months	1.2 (0.8,1.6)	1.7 (1.55,2.1)	t = -4.540, p = <0.0001

**Figure 5:**
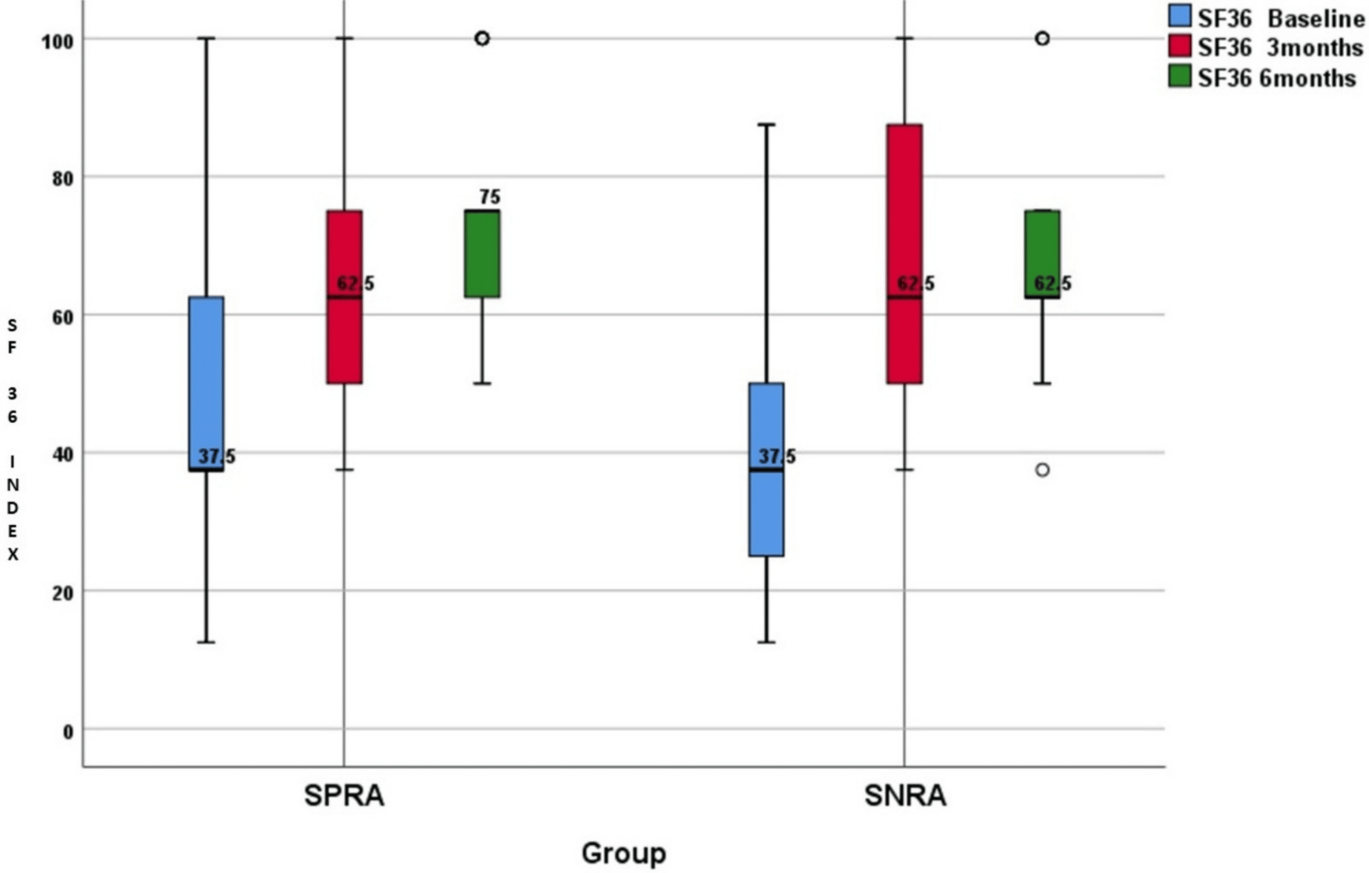
SF-36 Quality of life index of SPRA and SNRA at baseline, three months, and six months SF-36: 36-Item short form survey; SPRA: Seropositive rheumatoid arthritis; SNRA: Seronegative rheumatoid arthritis

**Figure 6:**
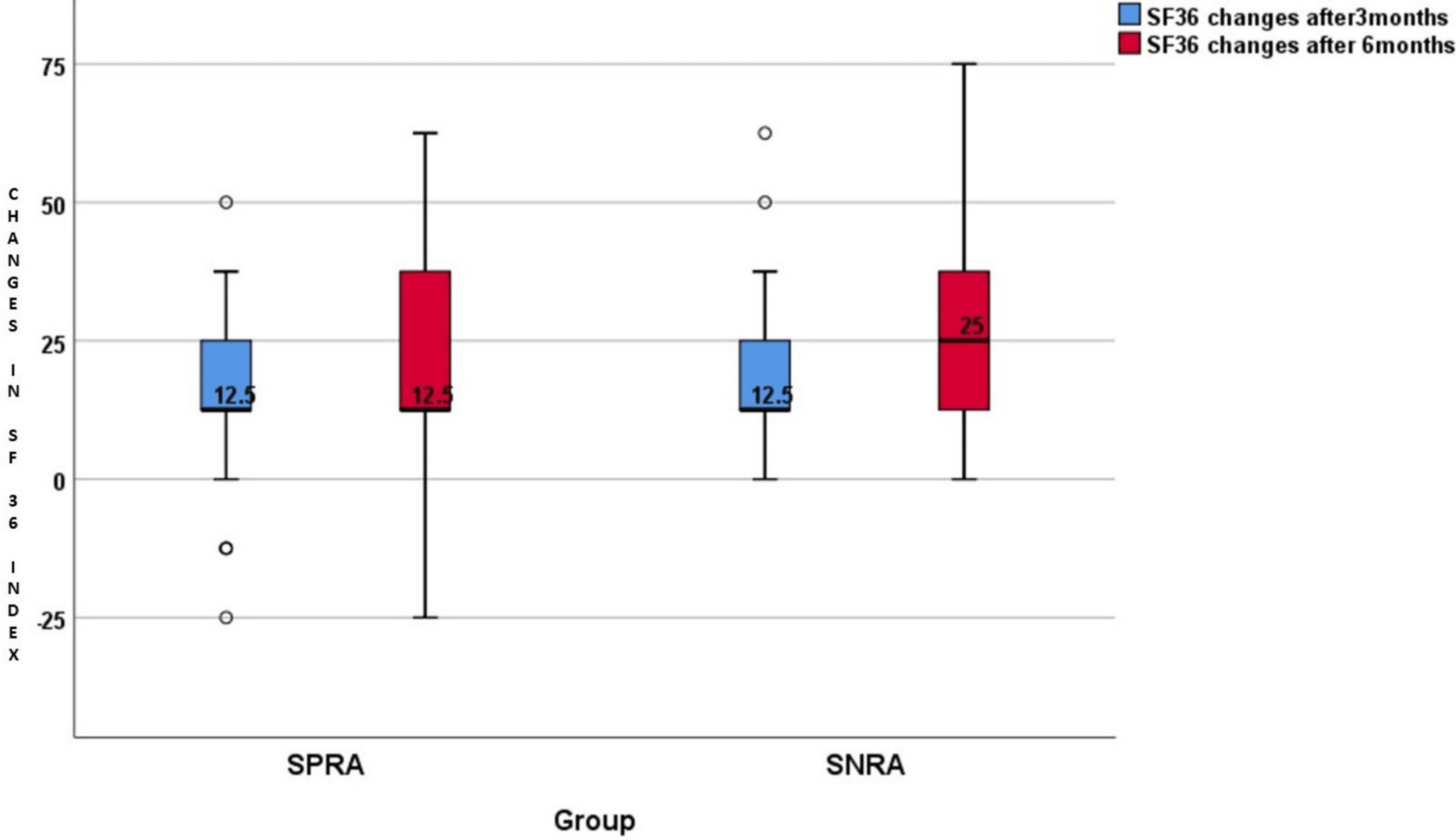
Changes in the SF-36 quality of life index after three months and after six months SF-36: 36-Item short form survey; SPRA: Seropositive rheumatoid arthritis; SNRA: Seronegative rheumatoid arthritis

The distribution of SF-36 index changes is summarised in Tables 4-7 and illustrated in Figures 5-6. The baseline values of the SF-36 index were comparable between the groups. The SF-36 index changed significantly in both groups when calculated after three months and after six months of initiation of csDMARDs (p<0.0001). While comparing SPRA and SNRA, the SF-36 index at baseline, three months, and six months for the groups was statistically similar (p>0.05). There were no statistical differences in SF-36 index changes between the two groups after three months (p=0.09) or after six months (p=0.08).

**Table 4:** SF-36 Quality of life index at baseline SF-36: 36-Item short form survey; SPRA: Seropositive rheumatoid arthritis; SNRA: Seronegative rheumatoid arthritis

SF-36 Index subscales at baseline	SPRA (n=87), median (IQR)	SNRA (n=17), median (IQR)	Test statistic & P-value
Physical Functioning (PF)	50 (25, 60)	40 (15, 60)	U = 653.5, p = 0.27
Role limitation-Physical (RP)	50 (0, 50)	0 (0, 62.5)	U = 676, p = 0.25
Role limitation-Emotional (RE)	66.7 (0, 100)	33 (0, 83.35)	U = 688.0, p = 0.3
Energy (E)	35 (20, 40)	40 (17.5, 52.5)	U = 739, p = 0.99
Mental Health (MH)	40 (36, 52)	44 (22, 62)	U = 699.5, p = 0.53
Social Function (SF)	37.5 (37.5, 62.5)	37.5 (25, 56.25)	U = 598, p = 0.14
Bodily Pain (BP)	32.5 (12.5, 45)	30 (10, 50)	U = 671.5, p = 0.28
Global Health (GH)	35 (25, 45)	25 (17.5, 50)	U = 722, p = 0.5
Health Change (HC)	25 (25, 50)	25 (0, 37.5)	U = 586.5, p = 0.08

**Table 5:** SF-36 Quality of life index at three months SF-36: 36-Item short form survey; SPRA: Seropositive rheumatoid arthritis; SNRA: Seronegative rheumatoid arthritis

SF-36 Index subscales at 3 months	SPRA (n=87), median (IQR)	SNRA (n=17), median (IQR)	Test statistic & P-value
Physical Functioning (PF)	55 (40, 65)	55 (25, 65)	U = 864.5, p = 0.79
Role limitation-Physical (RP)	50 (25, 75)	25 (25, 75)	U = 866.5, p = 0.49
Role limitation-Emotional (RE)	66.7 (33.3, 100)	66.7 (33.3, 100)	U = 851.5, p = 0.95
Energy (E)	55 (45, 60)	45 (35, 62.5)	U = 740, p = 0.53
Mental Health (MH)	56 (48, 64)	52 (46, 68)	U = 810, p = 0.545
Social Function (SF)	62.5 (50, 75)	62.5 (43.75, 87.5)	U = 902, p = 0.86
Bodily Pain (BP)	52.5 (40, 62.5)	50 (35, 62.5)	U = 861.5, p = 0.61
Global Health (GH)	55 (40, 60)	50 (35, 62.5)	U = 815.5, p = 0.61
Health Change (HC)	75 (50, 75)	50 (50, 75)	U = 923, p = 0.73

**Table 6:** SF-36 quality of life index at six months SF-36: 36-Item short form survey; SPRA: Seropositive rheumatoid arthritis; SNRA: Seronegative rheumatoid arthritis

SF-36 index subscales at 6 months	SPRA (n=87), median (IQR)	SNRA (n=17), median (IQR)	Test statistic & P-value
Physical Functioning (PF)	65 (50, 70)	70 (35, 77.5)	U = 710, p = 0.63
Role limitation-Physical (RP)	75 (50, 75)	50 (50, 75)	U = 723.5, p =0.81
Role limitation-Emotional (RE)	100 (66.7, 100)	100 (66.7, 100)	U = 740, p = 0.97
Energy (E)	60 (50, 65)	60 (50, 75)	U = 729, p = 0.86
Mental Health (MH)	56 (52, 64)	56 (54, 74)	U = 744.5, p = 0.99
Social Function (SF)	75 (62.5, 75)	62.5 (62.5, 87.5)	U = 742, p = 0.99
Bodily Pain (BP)	55 (45,67.5)	55 (45, 72.5)	U = 732, p = 0.92
Global Health (GH)	55 (50, 65)	55 (42.5, 67.5)	U = 743.5, p = 0.99
Health Change (HC)	75 (75, 75)	75 (50, 100)	U = 698, p = 0.83

**Table 7:** Distribution of SF-36 index changes SF-36: 36-Item short form survey; SPRA: Seropositive rheumatoid arthritis; SNRA: Seronegative rheumatoid arthritis

SF-36 Index	SPRA (n=87), median (IQR)	SNRA (n=17), median (IQR)	Test statistic & P-value
Baseline	37.5 (37.5, 62.5)	37.5 (25, 56.25)	U = 598, p = 0.14
3 months	62.5 (50, 75)	62.5 (43.75, 87.5)	U = 731, p = 0.86
6 months	75 (62.5, 75)	62.5 (62.5, 87.5)	U = 742, p = 0.99
SF-36 changes after 3 months	12.5 (12.5, 25)	12.5 (12.5, 25)	U = 645.5, p = 0.09
SF-36 changes after 6 months	12.5 (12.5, 37.5)	25 (12.5, 37.5)	U = 586.5, p = 0.08
P-Value (SF-36 changes after 3 months)	<0.0001	<0.0001	
P-Value (SF-36 changes after 6 months)	<0.0001	<0.0001	

## Discussion

This is a prospective comparative non-randomized observational study done for 16 months period. A total of 104 patients with RA were diagnosed according to the 2010 EULAR criteria. Patients were divided into two groups based on their seropositivity for SPRA and SNRA. We had 87 patients in the SPRA group and 17 patients in the SNRA group who were treated with csDMARDs, short course of glucocorticoids, NSAIDs, and exercise therapy.

The outcome measures in both groups were analysed with the VAS score, DAS28 ESR score, and SF-36. The VAS was used in many studies to assess the pain and functional and neurological status of the patients. The DAS28 ESR score was used to assess the severity of disease before and after starting csDMARDs. In our study, we have used the HRQoL index (SF-36), which is not extensively analyzed in patients of both SPRA and SNRA, except for a few studies.

In this study, the mean age of SPRA patients was 44.01±8.38 and that of SNRA patients was 46.23±9.99 (Table 1). This finding was in concordance with a cross-sectional study conducted by Mandal et al. in 2020 in a tertiary care hospital in Kolkata, who found that the mean age of RA patients was 43.1 years (mean age±SD: 43.05±10.63 years) [17].

We have also found that, in both SPRA and SNRA, there is a female predominance (Table 1). This is in concordance with previous studies on the prevalence of RA, which showed that women are affected by the disease approximately three times more than men. Doran et al. in 2002 conducted an incident cohort study of 609 patients with RA and found that 445 (73.1%) of them were female and 164 (26.9%) were male, in concordance with our study [18].

Mandal et al. in their study done in Kolkata found that the proportion of female subjects was 84.5%, with the female-male ratio of around 5.4:1, also in concordance with our studies [17].

Pain intensity measurement with the VAS revealed that SNRA patients had significantly more pain than SPRA during the initial presentation. Both groups had pain, which was moderate. SPRA patients had an average VAS score of 6, while SNRA patients had an average score of 7. Our data support earlier findings by Nordberg et al. in 2018, where they found that, at initial presentation, SNRA patients had a significantly higher VAS score (4.9) compared to SPRA patients (3.9) [9].

After the initiation of csDMARDs in both groups, VAS scores were measured at three and six months. It was found that SNRA patients responded better compared to SPRA after six months. The average VAS scores of SPRA and SNRA patients at three months were 3 and 5, respectively, and at six months were 3 and 3, respectively. Even though the pain of patients in both groups decreased from moderate to mild, the difference in the VAS scores after three and six months was significantly higher in the SNRA group (Table 2). This result was in concordance with the study done by Boer et al. in 2018, in which SNRA patients reported statistically significant more pain than SPRA patients at baseline. However, over the course of four years of the disease, both patient groups had equal amounts of pain [19]. Nordberg et al. in 2018 found, in their study, that the difference in the VAS scores after 24 months of follow-up was significantly higher in the SNRA group, which is also similar to our study [9].

DAS28 ESR scores of both SPRA and SNRA patients showed that, at baseline, SNRA had high disease activity with an average score of 5.2. At the same time, SPRA patients had medium disease activity, with an average score of 4.7. However, this finding was found to be statistically insignificant.

Choi et al. in 2018 found that, at baseline, DAS28 ESR (5.1±1.0 vs. 4.7±1.0; p = 0.043) were significantly higher in SNRA patients compared with those of SPRA patients [20]. Similarly, Barra et al. in 2014 in their Canadian Early Arthritis Cohort study found that DAS28 scores (5.0±1.6 vs. 4.8±1.5; p = 0.0493) of SNRA patients were significantly higher than those of SPRA patients at baseline [21].

After the initiation of DMARDs, both groups responded similarly at three months and six months, as evaluated by the DAS28 ESR scoring. DAS28 ESR scores of both groups significantly improved from the baseline. The scores of SPRA and SNRA patients at three months were 4 and 4.2, respectively, and at six months were 3.5 and 3.4, respectively.

Although the SNRA group had a higher disease activity at baseline compared to the SPRA group, the SNRA group showed a better treatment response, which was attributed to the similar treatment outcomes in the two groups at the end of six months of follow-up. The SNRA group had a significant reduction in DAS28 ESR scores compared to SPRA at three months and six months. The difference in DAS28 ESR scores at three months was 0.6 and 1 for SPRA and SNRA groups, respectively, which was statistically significant (p<0.0001). Similarly, the difference in DAS28 ESR scores at six months was 1.2 and 1.7 for SPRA and SNRA groups, respectively, which was also statistically significant (p<0.0001) (Table 3).

This result supports earlier findings done by Choi et al., who found that SNRA patients had a significantly higher difference in the DAS28 ESR score (ΔDAS28 from baseline at one year in SNRA -2.9±1.2 vs. SPRA -2.2±1.8; p = 0.002) compared to SPRA patients at the end of one year follow-up [20].

Similar results were reported in the study done by Barra et al. in 2014. At 12 months follow-up, the SNRA group had a larger mean decrease in the DAS28 ESR score (-2.1±1.9) compared to that of the SPRA group (-1.9±1.7). This difference in mean decrease remained significant at the 24-month follow-up. At the 24-month follow-up, the SNRA group had a mean decrease in the DAS28 ESR score of -2.4±2.0, and the SPRA group had a mean decrease of -1.8±1.8 with a p value of 0.0152 [21].

In this study, we found that HRQoL was negatively impacted by RA at baseline. We have used the SF-36 questionnaire to assess the physical and emotional well-being of the SPRA and SNRA groups.

There was a decrease in mean scores of all the parameters (physical functioning, role limitation-physical, role limitation-emotional, energy, emotional well-being, social function, bodily pain, general health, and health change) of the SF-36 QoL index in both groups at baseline. There were no significant differences in any of the parameters between SPRA and SNRA (Table 4). Similarly, Seegobin et al. in 2014 in their CARDERA trial found that the SF-36 physical component summary and SF-36 mental component summary were decreased in both SPRA and SNRA patients at baseline without any significant difference between them [22].

SF-36 assessment after three- and six-month follow-ups shows that the mean scores of all the parameters had improved in both groups (Tables 5-6). It was interesting to note that the parameters in the mental health domain showed a greater change than the physical health domain after three and six months in both groups. This may be due to the greater level of acceptance of the disease after experiencing symptoms for a long duration.

The difference in the SF-36 QoL index from baseline to three months and from baseline to six months was compared between the SPRA and SNRA groups. The SF-36 QoL index changes after three months in the SPRA group were 12.5 (12.5, 25), and those of the SNRA group were 12.5 (12.5, 25). The changes after six months in the SPRA group were 12.5 (12.5, 37.5), and those of the SNRA group were 25 (12.5, 37.5). This comparison between the two groups showed no significant statistical differences, neither during the three months (p=0.09) nor during the six months (p=0.08) (Table 7).

Similar results were reported in a comparative study done by Nordberg et al. in 2018. The comparison of the SF-36 physical components summary (PCS) and mental components summary (MCS) between the SPRA and SNRA groups showed no significant statistical difference. The SF-36 PCS score was 36.6±9.7 in the SPRA group and 32.5±7.9 in the SNRA group, with a p value of 0.37. The SF-36 MCS score was 49.3±10.3 in the SPRA group and 48.6±11.9 in the SNRA group, with a p value of 0.75 [9].

In this study, we studied the difference in HRQoL outcomes in the SPRA and SNRA patients. All our patients had moderate-to-severe disease at the time of presentation. We found that the SNRA group manifested a more severe disease at baseline compared to the SPRA group. This variation may be due to the use of the 2010 ACR/EULAR criteria for the diagnosis of RA patients. Since these criteria give more weightage to serologic markers for early detection of RA, most of the SNRA patients have more joint involvement compared to SPRA patients for fulfilling the diagnostic criteria.

However, we found that the SNRA group showed better response to treatment with csDMARDs than the SPRA group treated similarly. This was observed from the significant decrease in pain and disease severity shown by the SNRA group as measured by the VAS and DAS28 ESR scoring.

The better response to treatment did not reflect in the HRQoL assessment done by the SF-36 questionnaire. Both groups showed similar outcomes in both physical and mental health domains at baseline, three months, and six months follow-up.

Coming to the second objective of our study, we found that both groups showed significant improvement in QoL after the initiation of csDMARDs. Both SPRA and SNRA groups presented with a decreased SF-36 QoL index. This decrease was spread over all the individual parameters of the SF-36 index. This shows that RA presents as a significant burden both physically and mentally. After the initiation of csDMARDS, the SF-36 index values increased in both groups. The changes in the SF-36 QoL index were measured from baseline to three months and from baseline to six months, which were found to be statistically significant in both groups (p<0.0001). All our patients had a diagnosis of RA for less than one year and had not started with DMARDs. This made it favorable for the use of csDMARDs as the first-line treatment strategy. Both groups reported significant improvement in all the parameters of the SF-36 QoL index at three months and six months follow-up.

This result was in concordance with the randomized control trial done by Kosinski et al. in 2002, in which they found that all the SF-36 index subscales were decreased and significantly below normative values in early RA patients before treatment with methotrexate. After 52 weeks of treatment, they found out that the SF-36 index improved significantly across all subscales and physical and mental component summary measures [23].

This was also similar to the study done by Moreland et al. in 2012, who observed that RA patients treated with csDMARDs showed significant improvement in QoL and function as calculated by the modified Health Assessment Questionnaire (M-HAQ) after the three-year follow-up period. They also observed that there was a significant reduction in DAS28 ESR scores from 5.8±1.1 to 2.9±1.5 (p<0.0001) in the treatment group receiving csDMARDs triple therapy after the two-year follow-up period [24].

Korpela et al. in 2004 found in their study that there was a significant reduction in median DAS28 scores from 5.33 (IQR: 4.79, 6.04) to 2.00 (IQR: 1.32, 3.00) in the RA treatment group receiving csDMARDs triple therapy after the two-year follow-up period [25].

Möttönen et al. in 1999 observed in their study that early RA patients receiving csDMARDs triple therapy had a significant mean change of -28 in VAS scores after 24 months follow-up period [26].

Early diagnosis and treatment are often associated with good treatment outcomes in RA. The main advantage of using HRQoL measures such as the SF-36 questionnaire in the assessment of response to treatment is that these are patient-based measures. Patients report their own experiences and perceptions in the SF-36 questionnaire. This can help in the establishment of treatment regimen compliance, persistence, and cost-effectiveness.

Limitations

The present study has certain limitations. It was conducted at a single centre, which may restrict the generalizability of the findings. The sample size of the SNRA group was relatively small, which can increase the risk of type 2 error and potentially limit the strength of comparisons. Additionally, the follow-up periods of three and six months allowed only short-term outcomes to be evaluated, making it difficult to determine the long-term benefits of csDMARD therapy. Furthermore, most available literature comparing SPRA and SNRA and assessing HRQoL using the SF-36 originates from studies conducted outside India, resulting in a reliance on international references.

## Conclusions

We conclude that the initiation of csDMARDs in early rheumatoid arthritis patients results in significant improvement in QoL. They are an excellent initial treatment strategy for patients burdened by the disease. Both SPRA and SNRA patients showed significant improvement in QoL. This improvement was comparable between the groups without any significant differences. Though SNRA patients presented initially with increased pain and disease severity compared to the SPRA patients, they showed a more significant reduction in pain and disease severity than SPRA patients after the initiation of csDMARDs.
